# Supplementary material for: Contact Characteristics and Tribological Properties of the Weaving Surface of Mn-Cu and Fe-Zn Damping Alloys
Source: Materials (Basel). 2022 May 5;15(9):3303. doi: 10.3390/ma15093303 (PMC9105199; doi:10.3390/ma15093303)
Supplement: Supplementary file 1 [file materials-15-03303-s001.zip › materials-1652880-supplementary.pdf]

materials

Article

Contact Characteristics and Tribological Properties of the Weaving Surface of Mn-Cu and Fe-Zn Damping Alloys

Lin Zhang [1,*], Xindong Yan [1], Ying Shu [1], Hongjuan Yang [1], Xiaomin Kang [2], Zhenbing Cai [3] and Minhao Zhu [3]

1 School of Mechanical and Electrical Engineering, Chengdu University of Technology, Chengdu 610059, China; hpuxindong@163.com (X.Y.); shuying20000105@163.com (Y.S.); yhj1116@126.com (H.Y.)
2 School of Mechanical Engineering, University of South China, Hengyang 421001, China; kxmswjtu@163.com
3 Institute of Tribology, Southwest Jiaotong University, Chengdu 610031, China; czb_jiaoda@126.com (Z.C.); zhuminhao@139.com (M.Z.)
* Correspondence: zllz19891@163.com

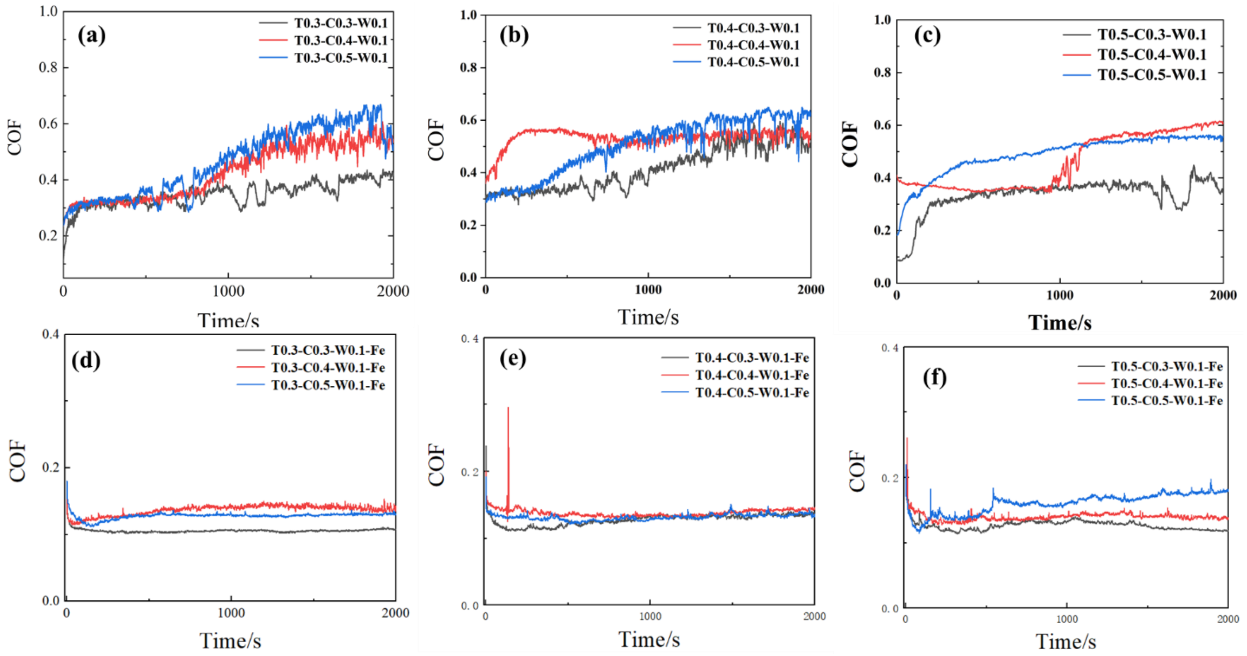

Figure S1. Friction coefficients of samples with different weaving parameters (**a**) Cofficient of Mn-Cu samples(T0.3-W0.1); (**b**) Cofficient of Mn-Cu samples(T0.4-W0.1); (**c**) Cofficient of Mn-Cu samples(T0.5-W0.1); (**d**) Cofficient of Fe-Zn samples(T0.3-W0.1); (**e**) Cofficient of Fe-Zn samples(T0.4-W0.1); (**f**) Cofficient of Fe-Zn samples(T0.5-W0.1).

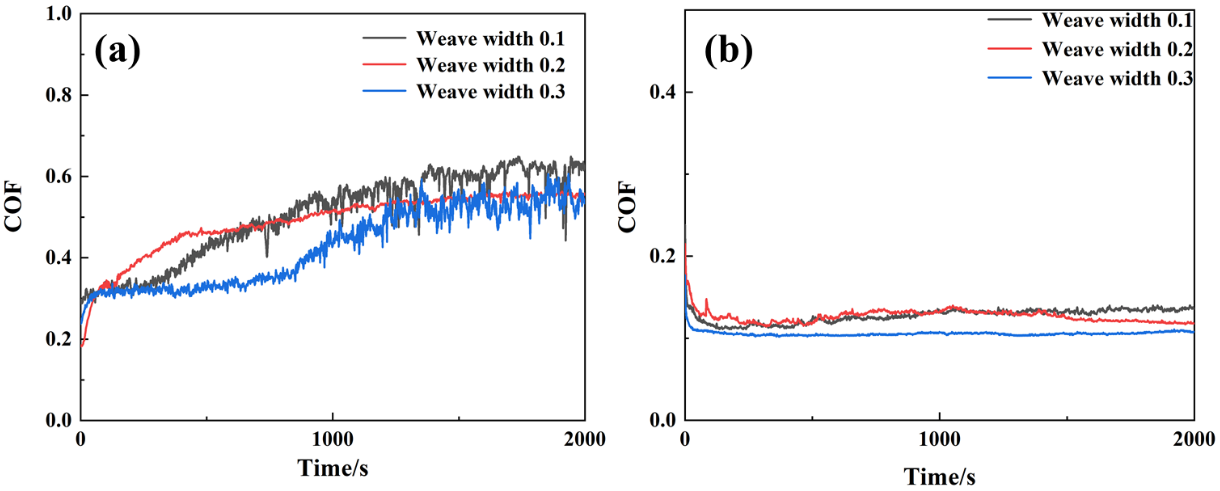

Figure S2. Friction coefficients of samples with different weaving width (**a**) Mn-Cu samples; (**b**) Fe-Zn samples.

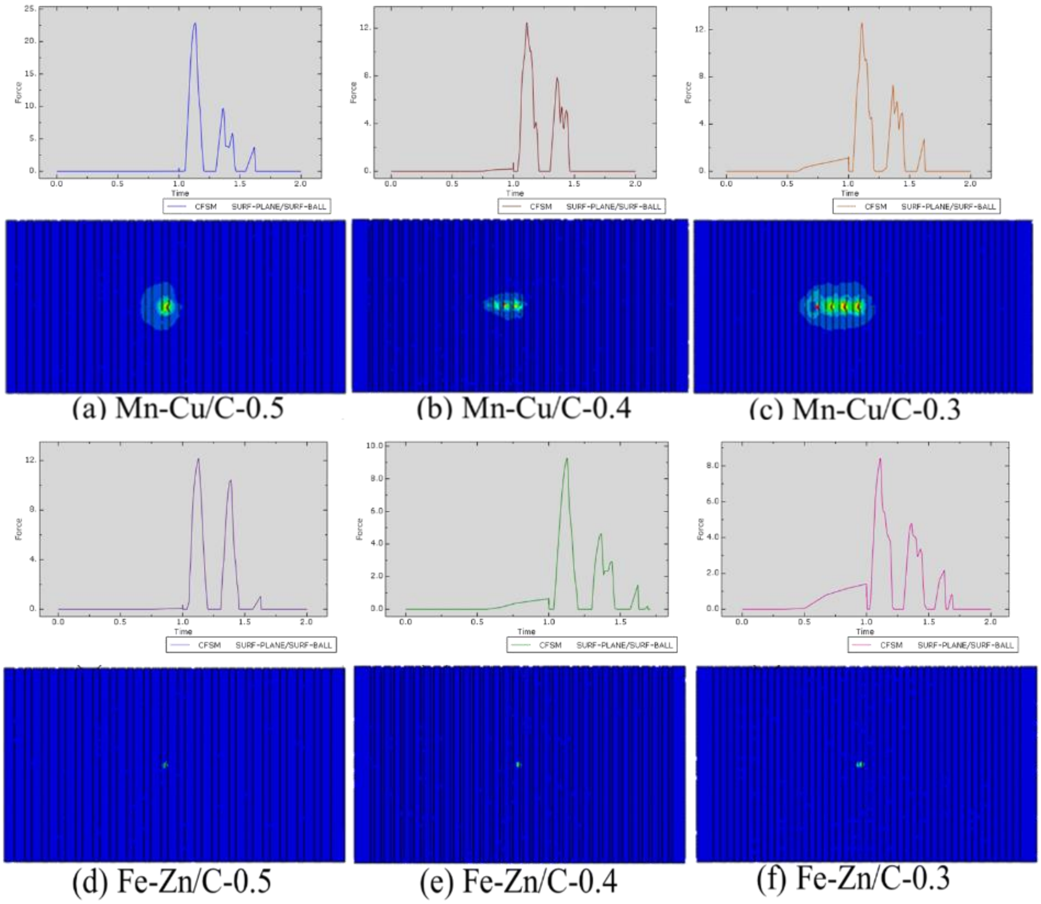

Figure S3. Friction process simulation of Mn-Cu samples with different weave center distance (**a**) C-0.5; (**b**) C-0.4; (**c**) C-0.3; and Fe-Zn samples with different weave center distance (**d**) C-0.5; (**e**) C-0.4; (**f**) C-0.3.

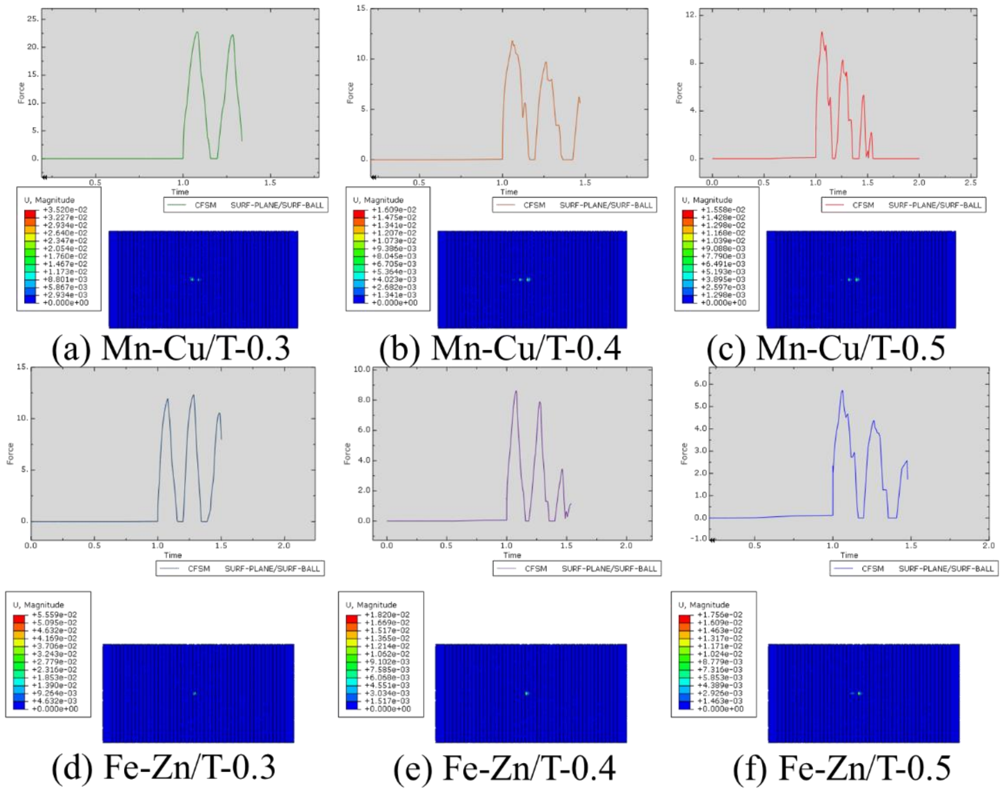

Figure S4. Friction process simulation of Mn-Cu samples with different thickness (**a**) T-0.5; (**b**) T-0.4; (**c**) T-0.3; and Fe-Zn samples with different thickness (**d**) T-0.5; (**e**) T-0.4; (**f**) T-0.3.

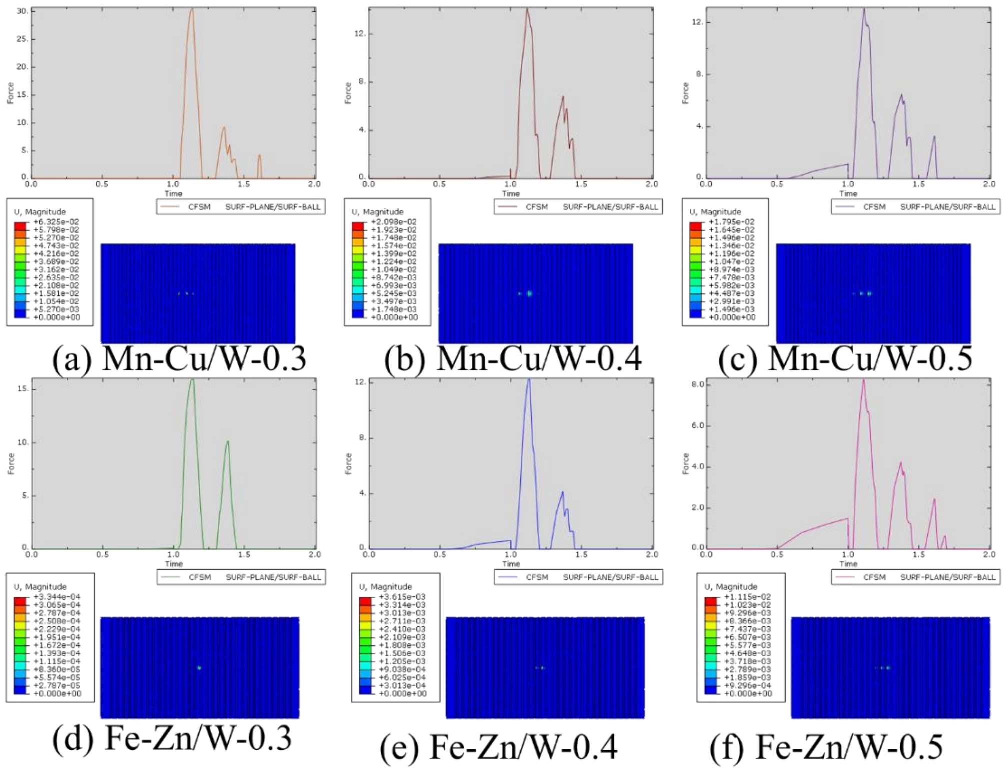

Figure S5. Friction process simulation of Mn-Cu samples with different weave width (**a**) W-0.5; (**b**) W-0.4; (**c**) W-0.3; and Fe-Zn samples with different weave width (**d**) W-0.5; (**e**) W-0.4; (**f**) W-0.3.
